# Incidence of Ventricular Fibrillation and Sustained Ventricular Tachycardia Complicating Non-ST Segment Elevation Myocardial Infarction †

**DOI:** 10.3390/jcm13082286

**Published:** 2024-04-15

**Authors:** Asher Schnur, Moshe Rav Acha, Ranel Loutati, Nimrod Perel, Louay Taha, Netanel Zacks, Tomer Maller, Mohammad Karmi, Feras Bayya, Nir Levi, Pierre Sabouret, Noam Fink, David Marmor, Mony Shuvy, Michael Glikson, Elad Asher

**Affiliations:** 1 Jesselson Heart Center, Shaare Zedek Medical Center, The Eisenberg R&D Authority, Faculty of Medicine, Hebrew University of Jerusalem, Jerusalem 9190500, Israel; ravacham@szmc.org.il (M.R.A.); ranellout@gmail.com (R.L.); n_perel@yahoo.co.in (N.P.); louayt@szmc.org.il (L.T.); netanelz@szmc.org.il (N.Z.); tomerma@szmc.org.il (T.M.); mkarmi@szmc.org.il (M.K.); bayya@szmc.org.il (F.B.); nir182@gmail.com (N.L.); davidma@szmc.org.il (D.M.); monysh@szmc.org.il (M.S.); mglikson@szmc.org.il (M.G.); easher@szmc.org.il (E.A.); 2 ACTION Study Group, Institut de Cardiologie, Hôpital Pitié-Salpêtrière, Sorbonne Université, 75005 Paris, France; cardiology.sabouret@gmail.com; 3 Department of Cardiology, National College of French Cardiologists, 13 Rue Niepce, 75014 Paris, France; 4 Assuta Medical Centers, Tel Aviv 6329302, Israel; noamfink@bezeqint.net; 5 Department of Military Medicine, Faculty of Medicine, Hebrew University of Jerusalem, Jerusalem 9190500, Israel

**Keywords:** ICCU, NSTEMI, arrhythmias, infarction, myocardial

## Abstract

**Background:** Primary ventricular fibrillation (VF) and sustained ventricular tachycardia (VT) are potentially lethal complications in patients suffering from acute myocardial infarction (MI). In contrast with the profound data regarding the incidence and prognostic value of ventricular arrhythmias in ST elevation myocardial infarction (STEMI) patients, data regarding contemporary non-ST elevation myocardial infarction (NSTEMI) patients with ventricular arrhythmias is scarce. The aim of the current study was to investigate the incidence of VF/VT complicating NSTEMI among patients admitted to an intensive coronary care unit (ICCU). **Methods:** Prospective, single-center study of patients diagnosed with NSTEMI admitted to ICCU between June 2019 and December 2022. Data including demographics, presenting symptoms, comorbid conditions, and physical examination, as well as laboratory and imaging data, were analyzed. Patients were continuously monitored for arrhythmias during their admission. The study endpoint was the development of VF/sustained VT during admission. **Results:** A total of 732 patients were admitted to ICCU with a diagnosis of NSTEMI. Of them, six (0.8%) patients developed VF/VT during their admission. Nevertheless, three were excluded after they were misdiagnosed with NSTEMI instead of posterior ST elevation myocardial infarction (STEMI). Hence, only three (0.4%) NSTEMI patients had VF/VT during admission. None of the patients died during 1-year follow-up. **Conclusions:** VF/VT in NSTEMI patients treated according to contemporary guidelines including early invasive strategy is rare, suggesting these patients may not need routine monitoring and ICCU setup.

## 1. Introduction

The number of patients diagnosed with non-ST elevation myocardial infarction (NSTEMI) is constantly rising and has surpassed those with ST elevation myocardial infarction (STEMI). This rise can be attributed to a multitude of factors, including an aging population, prolonged survival rates among individuals with coronary artery disease (CAD), and the increased sensitivity of troponin assays, which enables the earlier and more accurate diagnosis of NSTEMI in patients with a prior diagnosis of acute coronary syndrome (ACS) [1]. Ventricular fibrillation (VF) and sustained ventricular tachycardia (VT) are potentially life-threatening complications of acute myocardial infarction (MI), occurring in up to 5% of STEMI patients [1,2,3]. While these ventricular arrhythmias are well described among STEMI patients, they might also be a significant concern in the optimal management of NSTEMI patients [3,4]. Accordingly, current guidelines recommend continuous electrocardiogram (ECG) monitoring in the intensive coronary care unit (ICCU) for all NSTEMI patients [5,6,7,8]. However, while primary VF and VT are well-known predictors of mortality in STEMI and have been studied extensively over the years [9,10,11], data regarding the incidence and impact of VF/VT in contemporary NSTEMI patients are scarce. Several studies investigating the prevalence of VF/VT complicating NSTEMI have reported an incidence ranging between 1.5% and 4.6% [4,6,7,8]. However, these studies were often retrospective, conducted over a decade ago, and frequently included ventricular arrhythmias occurring during coronary intervention, which may have distinct pathophysiological mechanisms and prognostic implications [12,13,14]. Hence, this study aimed to prospectively investigate the incidence and prognostic significance of sustained VF/VT occurring during the hospitalization period (excluding ventricular arrhythmias during PCI procedures) of contemporary NSTEMI patients, who are routinely admitted to a tertiary care medical center ICCU and undergo early revascularization within 48 h of admission.

## 2. Methods

This prospective, single-center study spanned from June 2019 to December 2022.

Inclusion criteria were patients with a confirmed clinical, laboratory, and electrocardiographic diagnosis of NSTEMI upon presentation to the ICCU. The diagnosis of NSTEMI was based on symptoms of myocardial ischemia, new ECG ischemic changes, and a rising and/or falling pattern of high-sensitivity troponin with at least one value above the 99th percentile URL, according to the ESC guidelines for ACS. The diagnosis was made by the attending cardiologist. Moreover, all NSTEMI patients admitted to the ICCU were treated according to contemporary guidelines [15].

Exclusion criteria excluded patients with STEMI, unstable angina (UA), and out-of-hospital cardiac arrest (OHCA). The diagnosis of NSTEMI was determined based on elevated troponin levels coupled with ischemic symptoms, such as chest pain or angina equivalents, and specific ECG changes, including ST-segment depression or new-onset negative T-waves, aligning with contemporary guidelines.

All patients were continuously monitored for arrhythmias throughout the entire ICCU stay. Patient information was systematically recorded, and an electronic case report form (eCRF) was utilized for anonymous documentation and prospective submission of data upon admission to the ICCU. A local coordinator conducted regular checks for accuracy and flagged out-of-range values.

The study endpoints were defined as the development of VF or sustained VT during the ICCU admission, excluding arrhythmias occurring during percutaneous coronary intervention (PCI).

All patients with a diagnosis of NSTEMI within the timeframe mentioned were extrapolated, and all initial ECGs and coronary angiographies were systematically reviewed, and so 3 ECGs were found to have been STEMI (including occluded artery in the angiography) and not NSTEMI.

The ethical aspects of the study were addressed and approved by the Shaare Zedek Medical Center Institutional Review Board (IRB). Striving to maintain participants’ anonymity, the IRB approved de-identification during the subsequent database analysis, and informed consent requirements were waived due to the observational nature of the study. (Approval number SZMC-0320-22, approved on 21 November 2022).

No external funding was sought for the study, and all methodologies adhered strictly to relevant guidelines and regulations. Statistical analyses were performed comprehensively, describing characteristics in terms of numbers and percentages for categorical variables, and means ± standard deviations or median with interquartile ranges for continuous variables. Chi-square and Fisher’s exact tests were employed to evaluate relationships between categorical variables.

## 3. Results

### 3.1. Patients with NSTEMI

The study encompassed a comprehensive examination of 732 patients diagnosed with NSTEMI who were admitted to the ICCU. The median length of hospitalization at the ICCU was found to be 2 days, with a range spanning from 1 to 21 days. Among the NSTEMI patients, a substantial 81% underwent coronary angiography during their hospital stay. Within this subset, 76% opted for PCI, while 5% were referred for Coronary Artery Bypass Surgery (CABG) during their admission.

### 3.2. Patient’s Characteristics

The demographic characteristics of the patient cohort is detailed in Table 1. The mean age was 67 (±13), with a predominant male representation, constituting 77% of the cohort. Pre-existing comorbidities were prevalent, with 60% having hypertension, 62% with dyslipidemia, 43% diagnosed with diabetes mellitus (DM), 34% active smokers, and 41% having a history of prior CAD.

### 3.3. Complications during Admission

Complications during admission were carefully monitored and recorded. Of the 732 NSTEMI patients admitted to the ICCU, a subset experienced various complications, including 2.4% with shock, 3% with congestive heart failure exacerbation, 0.6% with stroke or transient ischemic attack, 0.8% with reinfarction, 0.1% with stent thrombosis, 2.6% with acute renal failure, 2.2% with significant bleeding, 1.2% requiring blood transfusions, and 1% developing sepsis.

### 3.4. Malignant Arrythmias

An intriguing facet of the study focused on malignant arrhythmias. While only six (0.8%) patients initially diagnosed with NSTEMI developed VF or sustained VT during their ICCU admission, a meticulous review revealed that three out of the six cases had a misdiagnosis of posterior STEMI rather than NSTEMI, prompting their exclusion from the study. Consequently, a slim 0.4% of patients with a definitive diagnosis of NSTEMI experienced VF or sustained VT during their ICCU stay. Notably, all three instances occurred after PCI, with each patient having multivessel disease evident on their coronary angiogram (as illustrated in Table 2). Further characterization revealed that two patients had reduced Left Ventricular (LV) function on echocardiogram, while one patient exhibited preserved LV function but with significant LV hypertrophy (septal diastolic thickness of 2.8 cm). Importantly, none of the three VF/VT patients died within one year of the NSTEMI diagnosis.

## 4. Discussion

This study shows three major findings regarding the occurrence of malignant arrhythmias in the context of NSTEMI. Firstly, our investigation revealed an exceptionally low incidence rate of malignant arrhythmias, standing at a mere 0.4%. Secondly, the manifestation of VF was identified as a rare phenomenon among conventional NSTEMI patients. Lastly, a noteworthy correlation emerged between the occurrence of VT/VF and specific cardiac conditions. All three patients who experienced VT/VF demonstrated multivessel disease during coronary angiography, coupled with either significantly diminished LV function or severe LV hypertrophy.

This study marks a significant contribution to the existing literature, as it stands, to the best of our knowledge, as the first prospective examination of VF/VT complications in contemporary NSTEMI patients admitted to a tertiary ICCU. This investigation focuses on a broad spectrum of patients, encompassing all-comers admitted to the ICCU and undergoing routine coronary angiography within 48 h from the onset of symptoms. The unique aspect of our study lies in its divergence from previous research that predominantly concentrated on the occurrence, risk factors, and prognostic implications of VF/VT in patients with STEMI [9,10,11]. While numerous studies have documented VF/VT in STEMI patients, the data regarding these malignant arrhythmias in NSTEMI patients have been notably scarce, often limited to sub-analyses or confined to single-center studies with selected cohorts undergoing early invasive treatment. Notably, a sub-analysis study derived from four randomized clinical trials reported an in-hospital VF/VT incidence of 2.1% [6], underscoring the infrequency of these events in the NSTEMI population. Another sub-analysis study yielded a slightly lower incidence of 1.5% of in-hospital VF/VT [7]. Additionally, two single-center studies showed a VF/VT incidence of 2.6% [4] and 4.3% [8] in NSTEMI patients. However, these studies were focused on examining risk factors associated with malignant arrhythmias such as platelet count and advanced age, but the incidence of VF/VT was not the primary endpoint. Moreover, these studies included ventricular arrhythmia events occurring during coronary intervention, which differs in pathophysiological mechanisms and prognostic significance. Furthermore, the temporal context of these prior studies cannot be overlooked, as they were predominantly conducted over a decade ago. During this period, NSTEMI patients were often admitted to internal medicine wards, with only the more critically ill ones being directed to the ICCU for continuous monitoring. This inherent bias may have led to an overestimation of ventricular arrhythmia incidence, as only high-risk NSTEMI patients were continuously monitored. Our study, in contrast, embraces a more contemporary setting, including all NSTEMI patients who were uniformly monitored within the ICCU and subjected to routine coronary angiography, with the majority undergoing revascularization within 48 h.

Notably, our findings challenge the prevailing notion by revealing a lower incidence of VF/VT relative to prior studies. This prompts us to question the imperative to admit all NSTEMI patients to the ICCU and may imply a more nuanced approach. Rather than a blanket admission strategy, our results suggest that mainly specific high-risk NSTEMI patients might derive greater benefit from continuous monitoring in ICCU setups. This insight into risk stratification could potentially revolutionize the management and allocation of resources for NSTEMI patients, emphasizing the need for a more personalized and targeted approach in clinical decision-making.

In our study, the remarkably low incidence of VF and VT can be attributed to various factors. For one, the results may reflect the successful implementation of guideline-directed management strategies within our tertiary medical center. Notably, a significant proportion of NSTEMI patients, comprising 81%, underwent coronary angiography, with 76% subsequently undergoing PCI and 5% being referred for CABG. This high adherence to guideline-recommended medical therapy, coupled with the prompt application of revascularization when indicated and the appropriate use of antiplatelet medications, likely played a pivotal role in mitigating the occurrence of ventricular arrhythmias.

Another contributing factor to the favorable outcomes observed in our cohort is the inclusion of all-comer NSTEMI patients, encompassing a diverse range of cases, including many relatively low-risk individuals. This stands in contrast to prior studies that predominantly focused on continuously monitoring sicker, high-risk patients. Remarkably, only 3% of our patients exhibited clear congestive heart failure (CHF) symptoms, and only 2.4% presented with cardiogenic shock, indicating that a substantial portion of our NSTEMI cases involved stable, low-risk patients. This deviation from previous studies, which reported much higher percentages of CHF patients (11% [7], 9.9% [3], and 6% [4] in various studies), underscores the unique composition of our study population and contributes to the overall positive outcomes observed. The inclusion of these stable, low-risk NSTEMI patients was a likely factor in the low percentage of ventricular arrhythmias in our study.

The current guidelines set forth by the European Society of Cardiology (ESC) [5] and the American Heart Association (AHA) [16] underscore the critical importance of monitoring and initiating timely invasive therapies for high-risk patients susceptible to adverse cardiovascular events, particularly ventricular arrhythmias. The latest guidelines from the ESC (2023) emphasize the need for prompt initiation of ECG monitoring in all patients with ACS [5] in order to allow for swift identification of life-threatening arrhythmias and facilitate timely defibrillation when necessary.

In alignment with these recommendations, the guidelines advocate for continuous ECG monitoring for at least 24 h after symptom onset in all high-risk ACS patients, which includes both STEMI patients and those with very high-risk NSTEMI (Class Ic) [5]. This stringent monitoring strategy aims to detect arrhythmias and new ST-segment elevation or depression, providing a crucial window for intervention and management.

The guidelines further suggest that prolonged monitoring may be considered for patients at intermediate to high risk of cardiac arrhythmias. This includes individuals who are hemodynamically unstable, present with major arrhythmias, have a left ventricular ejection fraction (LVEF) below 40%, experience failed reperfusion, exhibit additional critical coronary stenoses in major vessels, or encounter complications related to PCI.

In our study, we observed that the three NSTEMI patients who developed VT or VF all had multivessel coronary disease. Additionally, they exhibited markedly reduced LV function or severe LV hypertrophy, aligning with the guidelines that recommend prolonged continuous monitoring selectively for high-risk NSTEMI patients with such characteristics.

However, the study findings with extremely low incidence of VF/VT and the overall low death rate in these patients may prompt a reevaluation of monitoring approaches. There could be room for a more lenient monitoring strategy, especially in patients at lower risk—those with preserved LV function and single-vessel disease. Emphasizing the need for balancing between vigilant monitoring and the potential for over-monitoring and its associated resource implications.

Morality rate at 1 year was also low, and all three patients who suffered from VF/VT survived during the follow-up period. This absence of mortality in NSTEMI patients with ventricular arrhythmias is an encouraging finding, although the absolute number of patients with malignant arrhythmias was low.

## 5. Limitations

Despite the insights gained from our study, it is essential to acknowledge its limitations. First, being a single-center study might raise concerns about the generalizability of the findings. However, it is noteworthy that the study embraced a diverse group of participants in a real-world setting, enhancing the external validity of our observations. A second limitation pertains to the relatively modest number of patients experiencing ventricular arrhythmias, which could potentially constrain the precision of our conclusions regarding incidence rates and associated risk factors. Moreover, it is crucial to recognize that our study lacked the statistical power necessary for a comprehensive examination of long-term mortality in patients with arrhythmias following NSTEMI. Lastly, we do not have the time intervals regarding “pain—primary medical contact”, “pain—hospitalization” and “pain—PCI”.

## 6. Conclusions

VF and VT occurrences in NSTEMI patients undergoing treatment aligned with contemporary guidelines, which often involve early invasive strategies, are exceptionally rare. This infrequency underscores the critical importance of prompt recognition, appropriate admission, vigilant monitoring, and a tailored therapeutic approach to mitigate potential complications in these individuals. The findings suggest that in NSTEMI cases where the risk of mortality is not markedly high, monitoring NSTEMI outside the ICCU may be deemed reasonable, given the remarkably low incidence of VF/VT in this cohort. However, it is imperative to conduct further studies to comprehensively assess the long-term prognosis of such patients, to provide insights that can refine and optimize future management strategies for individuals experiencing NSTEMI.

## Figures and Tables

**Table 1:** Patients’ characteristics.

Baseline Characteristics	Total N = 729	NSTEMI without VF/VT N = 726	NSTEMI with VF/VT N = 3
Mean Age	66.8	66.8	70.3
Female	168 (23%)	168 (23%)	0 (0%)
Male	561 (77%)	558 (77%)	3 (100%)
Mean BMI (kg/m^2^)	28.14	28.14	28.7
HTN	484 (66%)	483 (66%)	1 (33%)
DLP	451 (62%)	448 (62%)	3 (100%)
DM	312 (43%)	310 (43%)	2 (67%)
Smoking	250 (34%)	249 (34%)	1 (33%)
Prior CAD	299 (41%)	297 (41%)	2 (67%)
Prior CVA	52 (7%)	51 (7%)	1 (33%)
PAD	44 (6%)	44 (6%)	0 (0%)
COPD/ASTHMA	38 (5%)	38 (5%)	0 (0%)
CHF	56 (8%)	54 (7%)	2 (67%)
CKD	81 (11%)	80 (11%)	1 (33%)
Malignancy	50 (7%)	50 (7%)	0 (0%)
Anemia	25 (3%)	23 (3%)	2 (67%)
A Fib./Flutter	48 (7%)	46 (6%)	2 (67%)
Prior CABG	75 (10%)	75 (10%)	0 (0%)

Abbreviations: NSTEMI—Non-ST Elevation Myocardial Infarction; VF/VT—Ventricular Fibrillation/Ventricular Tachycardia; BMI—Body Mass Index; HTN—Hypertension; DLP—Dyslipidemia; DM—Diabetes Mellitus; CAD—Coronary Artery Disease; CVA—Cerebral Vascular Accident; PAD—Peripheral Arterial Disease; COPD—Chronic Obstructive Pulmonary Disease; CHF—Congestive Heart Failure; CKD—Chronic Kidney Disease; A Fib.—Atrial Fibrillation; CABG—Coronary Artery Bypass Graft.

**Table 2:** VF/VT—patient characteristics and treatments.

Patient No.	Age (Years)	Sex	CHF Symptoms	Shock	Peak Troponin (ng/L)	Ejection Fraction (%)	Time from Symptom Onset to Coronary Angiography (Minutes)	PCI	Arrhythmia	Treatment of Arrhythmia
1	73	M	No	No	7376	50–54%	42	PCI to RCA and LCX	VT	Ablation
2	58	M	No	No	90,373	30–34%	139	Stent to RCA and LCX	VF	CPR DC ICD
3	80	M	No	No	240	35–39%	304	Stent to LM and LAD	VT	CPR ICD

Abbreviations: CHF—Congestive Heart Failure; PCI—Percutaneous Coronary Intervention; LM—Left Main; LAD—Left Anterior Descending; RCA—Right Coronary Artery; LCX—Left Circumflex; VT—Ventricular Tachycardia; PCI—Percutaneous Coronary Intervention; VF—Ventricular Fibrillation; CPR—Cardio-Pulmonary Resuscitation; DC—Direct Current Cardioversion; ICD—Implantable Cardioverter Defibrillator.

## Data Availability

The data presented in this study are available on request from the research institution (Shaare Zedek Medical Center).
